# Excess Long-Term Mortality following Non-Variceal Upper Gastrointestinal Bleeding: A Population-Based Cohort Study

**DOI:** 10.1371/journal.pmed.1001437

**Published:** 2013-04-30

**Authors:** Colin John Crooks, Timothy Richard Card, Joe West

**Affiliations:** 1 Division of Epidemiology and Public Health, The University of Nottingham, Nottingham City Hospital, Nottingham, United Kingdom; 2 Nottingham Digestive Diseases Centre, National Institute for Health Research Biomedical Research Unit, Queen's Medical Centre, Nottingham University Hospitals National Health Service Trust, Nottingham, United Kingdom; Barts and the London School of Medicine & Dentistry Queen Mary University of London, United Kingdom

## Abstract

**Background:**

It is unclear whether an upper gastrointestinal bleed is an isolated gastrointestinal event or an indicator of a deterioration in a patient's overall health status. Therefore, we investigated the excess causes of death in individuals after a non-variceal bleed compared with deaths in a matched sample of the general population.

**Methods and Findings:**

Linked longitudinal data from the English Hospital Episodes Statistics (HES) data, General Practice Research Database (GPRD), and Office of National Statistics death register were used to define a cohort of non-variceal bleeds between 1997 and 2010. Controls were matched at the start of the study by age, sex, practice, and year. The excess risk of each cause of death in the 5 years subsequent to a bleed was then calculated whilst adjusting for competing risks using cumulative incidence functions.

16,355 patients with a non-variceal upper gastrointestinal bleed were matched to 81,523 controls. The total 5-year risk of death due to gastrointestinal causes (malignant or non-malignant) ranged from 3.6% (≤50 years, 95% CI 3.0%–4.3%) to 15.2% (≥80 years, 14.2%–16.3%), representing an excess over controls of between 3.6% (3.0%–4.2%) and 13.4% (12.4%–14.5%), respectively. In contrast the total 5-year risk of death due to non-gastrointestinal causes ranged from 4.1% (≤50 years, 3.4%–4.8%) to 46.6% (≥80 years, 45.2%–48.1%), representing an excess over controls of between 3.8% (3.1%–4.5%) and 19.0% (17.5%–20.6%), respectively. The main limitation of this study was potential misclassification of the exposure and outcome; however, we sought to minimise this by using information derived across multiple linked datasets.

**Conclusions:**

Deaths from all causes were increased following an upper gastrointestinal bleed compared to matched controls, and over half the excess risk of death was due to seemingly unrelated co-morbidity. A non-variceal bleed may therefore warrant a careful assessment of co-morbid illness seemingly unrelated to the bleed.

*Please see later in the article for the Editors' Summary*

## Abstract 2

Colin Crooks and colleagues studied patient outcomes in the 5 years after a non-variceal bleed and found an increased risk of all causes of death, over half of which were due to non-gastrointestinal causes.

## Editors' Summary

**Background:**

Acute gastrointestinal bleeding is a potentially life-threatening abdominal emergency that remains a common cause of admission to hospital. Upper gastrointestinal bleeding (bleeding derived from a source above the ligament of Treitz, which connects the fourth portion of the duodenum to the diaphragm) is roughly four times as common as bleeding from the lower gastrointestinal tract and is a major cause of morbidity and mortality. Upper gastrointestinal bleeding is also relatively common—every year in the United States, roughly, 100,000 people are admitted to hospital for treatment for this acute medical emergency—and the most common cause is peptic ulcer disease. Inflammation and tears in the mucosa are also common causes.

**Why Was This Study Done?:**

In some acute emergencies, such as heart attacks (myocardial infarction), the most common cause of death relates to the associated cardiovascular disease but in other emergencies, such as stroke, death is commonly associated with other causes, such as cardiovascular disease and respiratory infections. However, despite upper gastrointestinal bleeding being the most common gastroenterology reason for admission to acute medicine, the long term outcomes of patients who have experienced a non-variceal upper gastrointestinal bleed are poorly understood (a variceal bleed refers to catastrophic bleeding from dilated collateral veins of the esophagus, usually seen in patients with cirrhosis of the liver). So in this study, in order to identify whether interventions might reduce mortality following a non-variceal upper gastrointestinal bleed (upper gastrointestinal bleed), the researchers used information from an English database to investigate the causes of death by age and time in the 5 years following such a bleed, and compared these results with deaths in a matched sample of the general population.

**What Did the Researchers Do and Find?:**

The researchers used longitudinal data from the English Hospital Episodes Statistics and General Practice Research Database to identify all patients who were admitted to the hospital for treatment of their first upper gastrointestinal bleed between April 1997 and August 2010. The researchers matched each case with a control of a similar age from the same General Practice and recorded causes of death from the whole cohort through the linked Office of National Statistics death register. The researchers then used a statistical method (cumulative incidence functions) to calculate the excess risk of death for those experiencing an upper gastrointestinal bleed compared to controls.

Using these methods, the researchers identified 16,355 people who had an upper gastrointestinal bleed, of whom 6,242 subsequently died, and 81,523 matched controls. The researchers found that the crude mortality rate in the first 5 years following an upper gastrointestinal bleed was 16.0 per 100 person**-**years. The rates and risk of death were 10%–15% lower for women than men. In the first month after a bleed, the mortality rate was increased for all causes of death, but the highest mortality rate was from non-malignant digestive disease (48 per 100 person**-**years), mostly linked to causes related to the upper gastrointestinal tract (35 per 100 person**-**years). For the remainder of the first year, the highest mortality rates were from malignant tumors (8.4 per 100 person**-**years), half of which were from sites outside the gastrointestinal tract. The researchers also found that circulatory and respiratory mortality rates were also increased over the first year but by 5 years, circulatory disease accounted for the highest mortality rate (2.5 per 100 person**-**years). In their analysis, the researchers calculated that the total 5-year risk of death due to gastrointestinal causes ranged from 3.6% in people ≤50 years to 15.2% in people ≥80 years, representing an excess risk of death of between 3.6% and 13.4%, respectively. The total 5-year risk of death due to non-gastrointestinal causes ranged from 4.1% in people ≤50 years to 46.6% in people ≥80 years, representing an excess risk of death of between 3.8% and 19.0%, respectively.

**What Do These Findings Mean?:**

These findings suggest that following an upper gastrointestinal bleed patients experienced excess risk of all causes of death, over half of which was due to non-gastrointestinal causes, particularly malignant tumors and cardiovascular disease. This excess in deaths was not explained by other simultaneous causes (co-morbidity) such as cancer or cardiovascular disease diagnosed before admission for the upper gastrointestinal bleed. An upper gastrointestinal bleed may be a marker of disease or an indicator of deterioration in non-gastrointestinal co-morbidity. A reassessment of non-gastrointestinal illnesses may be warranted in patients who have upper gastrointestinal bleeding.

**Additional Information:**

Please access these Web sites via the online version of this summary at http://dx.doi.org/10.1371/journal.pmed.1001437.

- Wikipedia provides information on upper gastrointestinal bleeding (note that Wikipedia is a free online encyclopedia that anyone can edit; available in several languages)
- MedlinePlus also provides useful information for patients on gastrointestinal bleeding
- The UK's NHS provides guidelines on managing acute gastrointestinal bleeding
- The Annals of Internal Medicine has published international consensus guidelines on managing non-variceal bleeding
- The journal Gut has published the Asia-Pacific consensus on managing non-variceal upper gastrointestinal bleeding

## Introduction

The causes of excess deaths following an acute medical event can demonstrate areas where mortality can be reduced. For example three-quarters of deaths following a myocardial infarction were found to be due to the cardiovascular disease itself, but after a stroke two-fifths of deaths were found to be due to related respiratory infections and cardiovascular disease [1],[2]. In contrast the long term outcomes of upper gastrointestinal haemorrhage are poorly understood, despite it being the most frequent cause of gastroenterology admission to acute medicine. Controlled studies have been limited to two cohorts with peptic ulcer disease from the early 1990s with fewer than 150 deaths [3],[4]. These showed an excess mortality unrelated to the bleeding event itself but the studies disagreed on which causes of death were increased. Other studies were uncontrolled [5],[6], smaller [7], not population based [8], or were so long ago as to be mostly irrelevant with respect to current management of bleeding [9]. Furthermore, an increasing proportion of non-variceal bleeds over the last two decades do not have underlying peptic ulcers, thereby reducing the relevance of these previous cause-of-death studies to current clinical practice [10].

Therefore, to identify whether interventions might reduce mortality following an upper gastrointestinal non-variceal bleed we investigated the causes of death by age and time in the 5 y following a non-variceal bleed, and compared them with deaths in a matched sample of the general population.

## Methods

### Data

A cohort study was designed using linked longitudinal data from the linked English Hospital Episodes Statistics (HES) data, General Practice Research Database (GPRD), and Office of National Statistics death register. This data linkage records all primary care events, hospital admissions, and causes of death between 1st April 1997 to 31st August 2010 for 3% of the English population. Because of the comprehensive English primary care system the population registered to the GPRD is representative of the general English population [11]. The data sources are subject to quality checks and a practice's data is only used when it is of high enough quality to be used in research [12]. This is referred to as the up to research standard time period and is defined separately for each primary care practice. Ethical approval for this study was obtained from the Independent Scientific Advisory Committee for Medicines and Healthcare products Regulatory Agency (MHRA) database research.

### Cohort

#### Population

We selected as exposed all patients with a first non-variceal upper gastrointestinal bleed. A bleed was defined by a specific code for an upper gastrointestinal non-variceal bleed in either primary or secondary care who had a supporting code in the linked dataset. We previously described and published this methodology [13]. All patients in the study therefore had a hospital admission at the time of their bleed, reflecting national guidelines at the time of the study [14]. Variceal bleeds or non-specific gastrointestinal bleed codes with either a lower gastrointestinal diagnosis or procedure were excluded. Further exclusions were temporary patients, children under 16 y old, cases with invalid date codes, or cases outside the up to research standard observed time periods. The observed up to research standard time periods began at the latest of: the start of the linked data (1st April 1997), the date the patient registered with a practice, or the up to research standard date of that practice. It ended at the earliest of: the last date for the linked primary care data (31st August 2010), the date a patient transfered out of care of the practice, a patient's death, or the last date of data collection from that practice.

Patients who had a bleed recorded prior to the observed up to standard time period of our database, either in the hospital admissions database or in the longitudinal primary care record, were excluded. Bleeds prior to the observed up to research standard time period should be captured by the longitudinal primary care record, as historical records would be transferred with a patient when they change their primary care provider. Patients were required to be registered with a new primary care practice for at least 3 mo prior to any upper gastrointestinal bleed event to avoid including prevalent cases that might have been coded at the initial registration consultation. Follow-up started on the day of the first bleed.

#### Comparison group

For each case five age- (±5 y) and sex-matched controls were selected who were alive at the time of the bleed and registered to the same general practice. Controls were required to have been registered with the primary care practice for at least 3 mo prior to the match date to be consistent with the definition for cases.

#### Causes of death

Dates of death for the whole cohort were extracted from the linked data using the Office of National Statistics death register. All deaths in England are coded and recorded in the Office of National Statistics Death register from death certificates using the World Health Organization (WHO) guidelines [15]. These define causes of death by International Classification of Diseases version 2010 (ICD 10) codes with the main underlying cause established for each death using standardised rules. For this study we analysed the underlying cause of death by the most frequent ICD 10 chapter headings of neoplasms (ICD chapters C and D), circulatory (ICD chapter I: including cerebrovascular and ischaemic heart disease), respiratory (ICD chapter J), digestive disease (ICD chapter K), and the remaining less frequent chapter headings grouped together in an “other causes” category. Neoplasms were further subdivided between upper gastrointestinal malignancies and other neoplasms. Causes of death prior to 2001 were coded using ICD 9 and were therefore mapped onto the relevant ICD 10 chapter headings.

#### Follow-up

Patients were followed up from the date of gastrointestinal bleed or matching until either death or censoring of the patient record (defined as the end of the observed up to research standard time period). Follow-up did not stop if a subsequent bleed occurred but continued until death or censoring of the patient record.

### Analysis

#### Crude mortality rates

Crude numbers of deaths and rates per 1,000 person**-**years following upper gastrointestinal bleed were calculated overall and by the most frequent ICD 10 chapter headings. These rates were then stratified by age group and year post bleed. Age was grouped into <50, 50–59, 60–69, 70–79, and ≥80-y-old. The time post bleed was stratified into the first 30 d, 1 mo to 1 y, and 1 y to 5 y.

#### Adjusted analysis

Crude mortality rates were calculated for those still alive and at risk at each time point. However, when studying specific causes of death this group of survivors might not be representative of the initial cohort, since deaths from other causes can select out those with relevant risk factors. One method to adjust for this bias uses cumulative incidence functions (CIFs) that calculate the probability of overall survival from all causes, combined with the instantaneous hazard of death for each specific cause [16]. CIF were therefore calculated for each cause of death using baseline survival functions and hazard ratios from Cox proportional hazards modelling. The models were stratified by age group, adjusted for gender, and split at 1 mo, 1 y, 3 y, and 5 y. The excess risk was calculated as the difference between the CIF for cases exposed to a bleed and the CIF for unexposed controls. 95% CIs were derived by bootstrapping (500 iterations).

#### Sensitivity analyses

We assessed whether the excess mortality associated with a bleed for each cause of death was confounded by pre-existing co-morbidity, excess alcohol, or smoking status, and whether it varied by the site of bleed. Pre-existing co-morbidity was measured by the Charlson index (a weighted co-morbidity score predicting 1 y mortality [17]) using both hospital and primary care records prior to 2 mo before the bleeding episode. Smoking and alcohol status for each patient was derived from the information available in the linked dataset. Smoking status was categorised as current smoker or non-smoker. Excess alcohol status was categorised as excess alcohol use (including consumption over the recommended limit, alcohol dependency codes, complications from chronic alcohol abuse, or therapy for alcohol dependency) or no excess alcohol use. Site of bleed was categorised as oesophageal, gastric, duodenal, or unspecified.

## Results

16,355 unique individuals who had a non-variceal upper gastrointestinal bleed were identified in the linked primary and secondary care dataset, with 6,242 subsequent deaths. 8 cases (0.05%) could not be matched to controls and were therefore excluded from the study. Baseline demographics are shown for the bleed cases and the matched controls in Table 1 along with the numbers of deaths for each of the ICD 10 chapter headings. For clarity of presentation in the remainder of the results, deaths not attributed to one of the most frequent ICD 10 chapter headings were grouped together as “other causes.” More than 70% of the ICD 10 “external” chapter causes of death were coded as either a fall, strangling, or unspecified exposure. More than 95% of the ICD 10 “symptoms” chapter causes were coded as senility. More than 70% of the ICD 10 “musculoskeletal” chapter causes were coded as either osteoporosis with fracture, rheumatoid arthritis, osteomyelitis, or pyogenic arthritis. More than 70% of the ICD 10 “dermatological” chapter causes of death were coded as ulcers or cellulitis. The overall median follow-up time from index date was 3.2 y (interquartile range 0.4–5.2), and for those who were censored without death was 4.8 y.

**Table 1:** Numbers, deaths, and follow-up time by exposure to upper gastrointestinal bleeding within 5 y of bleeding.

Characteristics	Exposed	Percent	Unexposed	Percent
**Cohort (** ***n*** **)**	16,355	—	81,523	—
Deaths	6,424	—	11,643	—
Person-years	40,137	—	274,043	—
**Gender (** ***n*** ** = patients)**				
Male	8,800	53.8	43,836	53.8
Female	7,555	46.2	37,687	46.2
**Age (** ***n*** ** = patients)**				
<60 y	4,698	28.7	24,009	29.5
60–69 y	2,512	15.4	13,223	16.2
70–79 y	4,178	25.5	22,110	27.1
≥80 y	4,967	30.4	22,181	27.2
**Number of deaths (percentage shown of all deaths)**				
Neoplasms	1,948	30.3	2,615	22.5
Circulatory	1,704	26.5	4,443	38.2
Digestive	1,042	16.2	390	3.3
Respiratory	787	12.3	1,724	14.8
Genitourinary	138	2.1	265	2.3
Psychiatric	119	1.9	398	3.4
Neurological	110	1.7	321	2.8
Infections	99	1.5	122	1.0
External	88	1.4	228	2.0
Symptoms	80	1.2	346	3.0
Endocrine	77	1.2	158	1.4
Musculoskeletal	49	0.8	95	0.8
Dermatological	27	0.4	35	0.3
Haematological	26	0.4	19	0.2
Poisoning	13	0.2	9	0.1
Congenital	6	0.1	6	0.1
Unassigned code	7	0.1	7	0.1
Uncoded	104	1.6	462	4.0

Out of a total of 1,536 deaths within 30 d of a bleed, 306 (17%) had an underlying cause coded as an upper gastrointestinal bleed determined by the following ICD 10 codes: gastrointestinal haemorrhage K922 (*n = *133), chronic duodenal ulcer with haemorrhage K264 (*n = *92), chronic gastric ulcer with haemorrhage K254 (*n = *44), and chronic peptic ulcer with haemorrhage K274 (*n = *27). The combined codes of chronic duodenal ulcer with haemorrhage and perforation K266, acute duodenal ulcer with haemorrhage K260, oesophageal haemorrhage K228, acute gastric ulcer with haemorrhage K250, and chronic gastric ulcer with haemorrhage and perforation K256 were the underlying cause in a further ten deaths.

### Crude Mortality Rates

The crude mortality rate in the first 5 y following an upper gastrointestinal bleed was 16.0 per 100 person**-**years, 95% CI 15.6–16.4. This number declined over time from 35.7 deaths per 100 person**-**years (95% CI 34.7–36.8) in the first year to 7.3 deaths per 100 person**-**years (95% CI 7.0–7.7) over the subsequent 4 y. The rates and risk of death were 10%–15% lower for women than men, but the relative differences between causes of death were similar. Therefore Table 2 shows the numbers of deaths and crude rates by ICD 10 category stratified by time post bleed. In the first month after a bleed the mortality rate was increased for all causes of death, but the highest mortality rate was from non-malignant digestive disease (48 deaths per 100 person**-**years), and this was mostly due to causes related to the upper gastrointestinal tract (35 per 100 person**-**years). For the remainder of the first year the highest mortality rates were from neoplasms (8.4 per 100 person**-**years), half of which were from sites outside the gastrointestinal tract. Circulatory and respiratory mortality rates were also increased over the first year, but to a lesser extent than for digestive disease and neoplasms. However, by 5 y the category with the highest mortality rate was circulatory disease (2.5 per 100 person**-**years). The mortality rates for each cause of death remained slightly higher at 5 y following an upper gastrointestinal bleed than in the matched controls (Table S1).

**Table 2:** Mortality rate per 100 person-years, stratified by cause of death by ICD10 headings in the 5 y post bleed.

Cause of Death	1st month Deaths (*n*)	Rate (95% CI)	1 mo to 1 y Deaths (*n*)	Rate (95% CI)	1 y to 5 y Deaths (*n*)	Rate (95% CI)
**Neoplasms** a	**521**	**41.3** (37.9–45.0)	**920**	**8.4** (7.8–8.9)	**507**	**1.8** (1.7–2.0)
Oesophagus^b^	85	6.7 (5.5–8.3)	151	1.4 (1.2–1.6)	53	0.2 (0.1–0.2)
Stomach	61	4.8 (3.8–6.2)	152	1.4 (1.2–1.6)	52	0.2 (0.1–0.2)
Colon	21	1.7 (1.1–2.6)	37	0.3 (0.2–0.5)	35	0.1 (0.1–0.2)
Pancreas	37	2.9 (2.1–4.1)	66	0.6 (0.5–0.8)	19	0.1 (0.0–0.1)
Digestive (other)	39	3.1 (2.3–4.2)	58	0.5 (0.4–0.7)	46	0.2 (0.1–0.2)
Respiratory	50	4.0 (3.0–5.2)	88	0.8 (0.6–1.0)	75	0.3 (0.2–0.3)
Skin or bone	12	1.0 (0.5–1.7)	18	0.2 (0.1–0.3)	9	0.0 (0.0–0.1)
Breast	28	2.2 (1.5–3.2)	27	0.2 (0.2–0.4)	21	0.1 (0.0–0.1)
Prostate	34	2.7 (1.9–3.8)	55	0.5 (0.4–0.7)	43	0.2 (0.1–0.2)
Other or benign	165	13.1 (11.2–15.3)	276	2.5 (2.2–2.8)	161	0.6 (0.5–0.7)
**Circulatory**	**378**	**30.0** (27.1–33.2)	**621**	**5.6** (5.2–6.1)	**705**	**2.5** (2.3–2.7)
Rheumatic disease	≤5	—	≤5	—	17	0.1 (0.0–0.1)
Hypertensive disease	≤5	—	6	0.1 (0.0–0.1)	15	0.1 (0.0–0.1)
IHD	134	10.6 (9.0–12.6)	209	1.9 (1.7–2.2)	292	1.0 (0.9–1.2)
Pulmonary circulatory disease	20	1.6 (1.0–2.5)	12	0.1 (0.1–0.2)	16	0.1 (0.0–0.1)
Heart - other	50	4.0 (3.0–5.2)	104	0.9 (0.8–1.1)	113	0.4 (0.3–0.5)
CVA	83	6.6 (5.3–8.2)	197	1.8 (1.6–2.1)	197	0.7 (0.6–0.8)
Other circulatory	47	3.7 (2.8–5.0)	48	0.4 (0.3–0.6)	42	0.2 (0.1–0.2)
**Respiratory**	**189**	**15.0** (13.0–17.3)	**302**	**2.7** (2.5–3.1)	**296**	**1.1** (0.9–1.2)
Respiratory infections	86	6.8 (5.5–8.4)	130	1.2 (1.0–1.4)	119	0.4 (0.4–0.5)
Chronic airway disease	48	3.8 (2.9–5.1)	71	0.6 (0.5–0.8)	108	0.4 (0.3–0.5)
ILD	16	1.3 (0.8–2.1)	23	0.2 (0.1–0.3)	22	0.1 (0.1–0.1)
Respiratory - other	39	3.1 (2.3–4.2)	78	0.7 (0.6–0.9)	47	0.2 (0.1–0.2)
**Digestive**	**608**	**48.2** (44.6–52.2)	**258**	**2.3** (2.1–2.7)	**176**	**0.6** (0.5–0.7)
Upper GI	436	34.6 (31.5–38.0)	96	0.9 (0.7–1.1)	43	0.2 (0.1–0.2)
Lower GI	80	6.3 (5.1–7.9)	52	0.5 (0.4–0.6)	51	0.2 (0.1–0.2)
Liver or gallbladder	82	6.5 (5.2–8.1)	100	0.9 (0.7–1.1)	74	0.3 (0.2–0.3)
Pancreas	6	0.5 (0.2–1.1)	8	0.1 (0.0–0.1)	8	0.0 (0.0–0.1)
**Other**	**171**	**13.6** (11.7–15.8)	**329**	**3.0** (2.7–3.3)	**339**	**1.2** (1.1–1.4)
**Uncoded**	**45**	**3.6** (2.7–4.8)	**38**	**0.3** (0.3–0.5)	**21**	**0.1** (0.0–0.1)
**Total**	**1,912**	**151.7** (145.1–158.7)	**2,468**	**22.4** (21.6–23.4)	**2,044**	**7.3** (7.0–7.7)

Due to anonymisation numbers in cells with five or fewer events are not shown.

a Bold headings indicate ICD 10 chapter headings.

b Non-bold headings indicate ICD 10 subchapter headings.

CVA, cerebrovascular accident; GI, gastrointestinal; IHD, ischaemic heart disease; ILD, interstitial lung disease.

The mortality rates for each of the causes of death increased with age except for the mortality rate from liver disease, which decreased with age (Table S2). The mortality rates were higher for each age group following an upper gastrointestinal bleed than for the matched controls (Table S3). Mortality rates were high and fell rapidly over the first year, therefore Tables S2 and S3 only show mortality rates between 1 and 4 y following the bleed, when the mortality rates were more stable. The highest mortality rate in the younger age groups was from neoplasms and digestive disease, whereas in older age groups the highest mortality rates were from circulatory disease, comprising mainly ischaemic heart disease (3.2 per 100 person-years) and cerebrovascular disease (3.3 per 100 person-years).

### Adjusted Analysis

**Figure 1:**
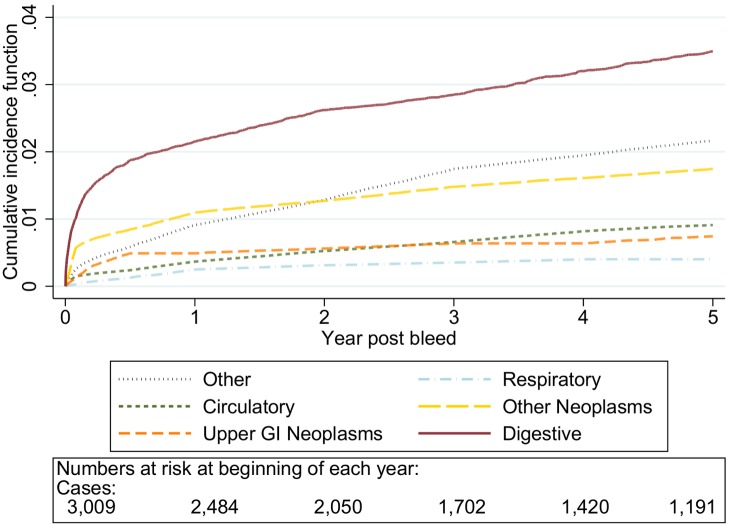
Cumulative incidence function for each cause of death following non-variceal bleeding ≤50 y. Cause of death by ICD 10 chapter over 5 y follow-up following a bleed. Numbers at risk at beginning of each year of follow-up shown beneath legend. Adjusted for gender and competing risks.

**Figure 2:**
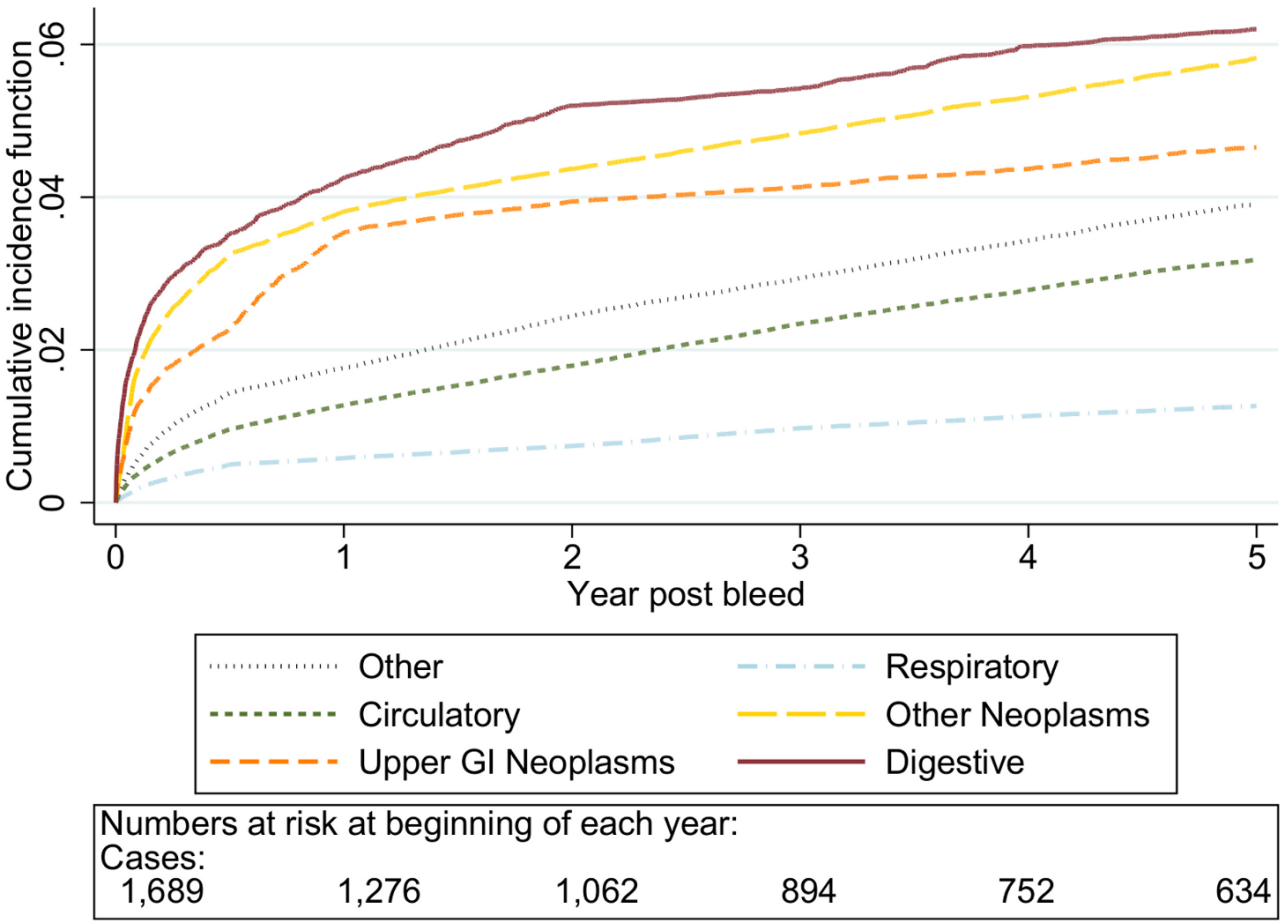
Cumulative incidence function for each cause of death following non-variceal bleeding 50–59 y. Cause of death by ICD 10 chapter over 5 y follow-up following a bleed. Numbers at risk at beginning of each year of follow-up shown beneath legend. Adjusted for gender and competing risks.

**Figure 3:**
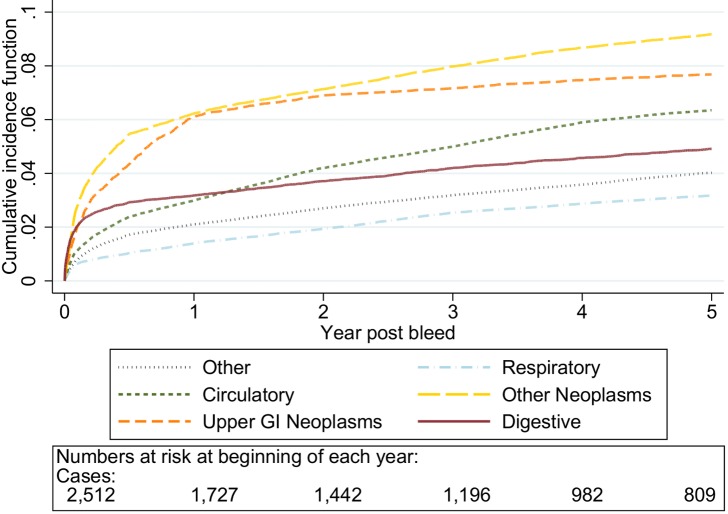
Cumulative incidence function for each cause of death following non-variceal bleeding 60–69 y. Cause of death by ICD 10 chapter over 5 y follow-up following a bleed. Numbers at risk at beginning of each year of follow-up shown beneath legend. Adjusted for gender and competing risks.

**Figure 4:**
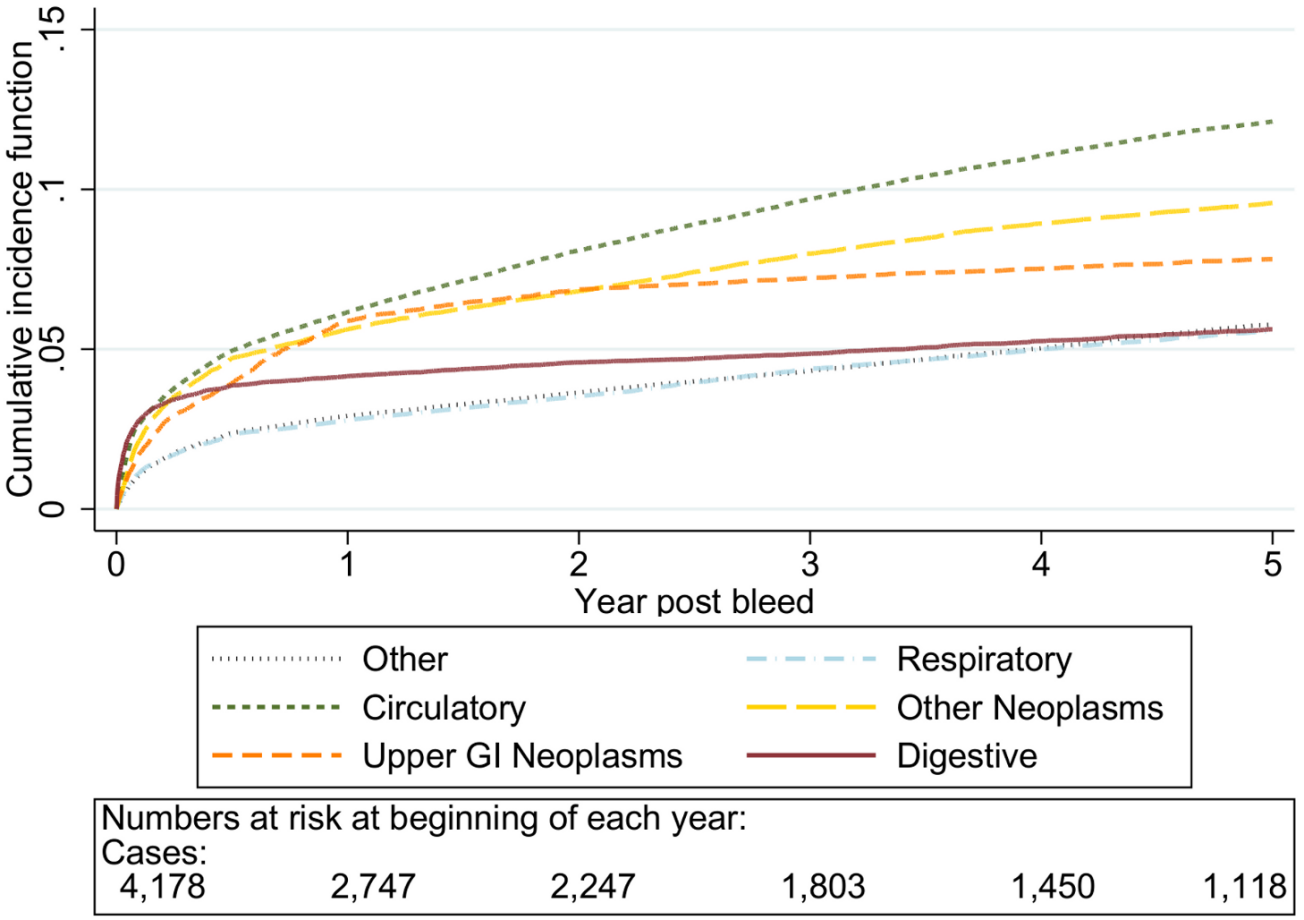
Cumulative incidence function for each cause of death following non-variceal bleeding 70–79 y. Cause of death by ICD 10 chapter over 5 y follow-up following a bleed. Numbers at risk at beginning of each year of follow-up shown beneath legend. Adjusted for gender and competing risks.

**Figure 5:**
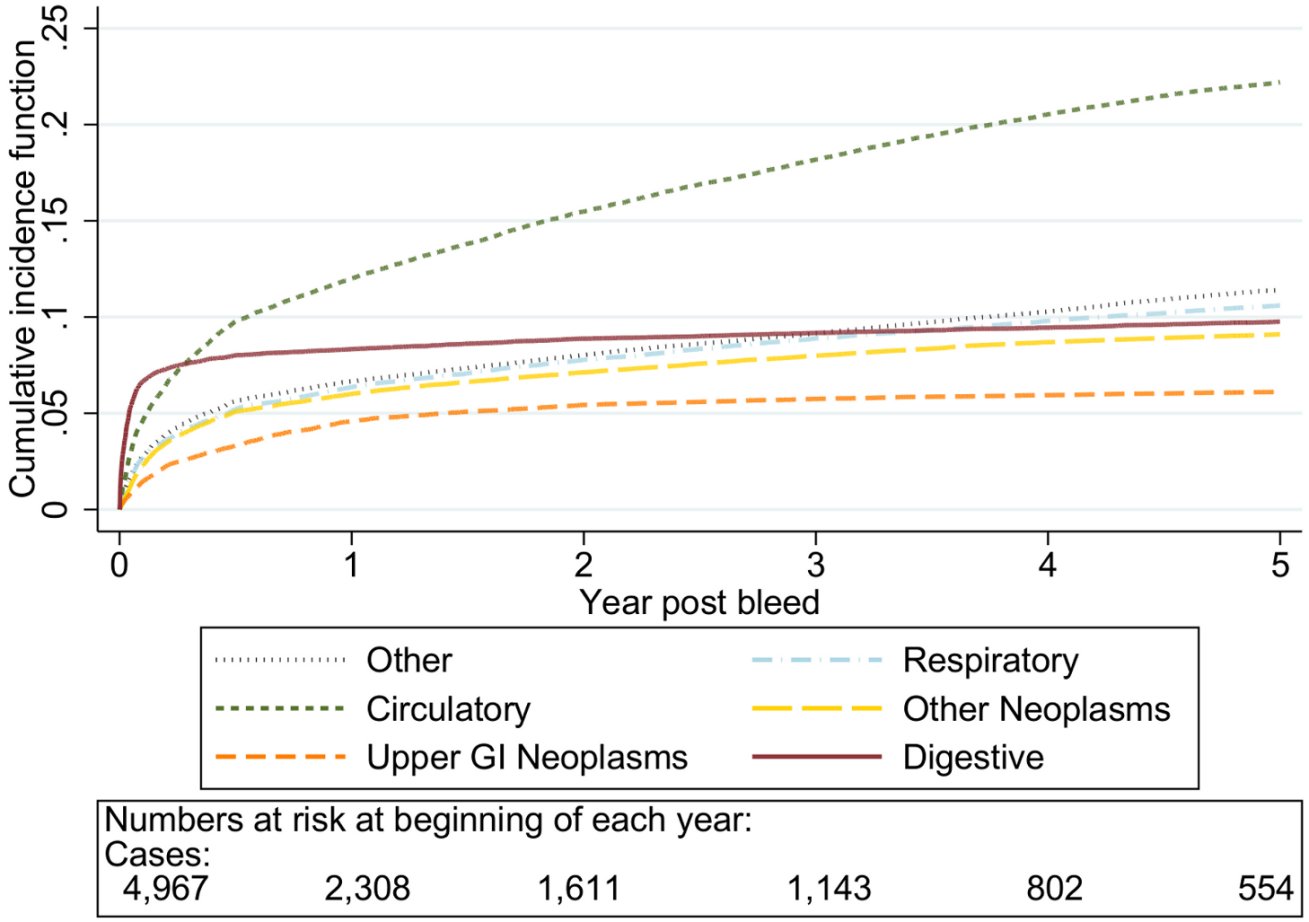
Cumulative incidence function for each cause of death following non-variceal bleeding ≥80 y. Cause of death by ICD 10 chapter over 5 y follow-up following a bleed. Numbers at risk at beginning of each year of follow-up shown beneath legend. Adjusted for gender and competing risks.

The graphs in Figures 1–5 show the CIF adjusted for competing risks for the most frequent causes of death by ICD 10 chapter headings stratified by age group. By 5 y after an upper gastrointestinal bleed the cumulative risk of death due to malignant or non-malignant gastrointestinal causes ranged from 3.6% (≤50 y, 95% CI 3.0%–4.3%) to 15.2% (≥80 y, 95% CI 14.2%–16.3%). In contrast the CIF for death due to non-gastrointestinal causes ranged from 4.1% (≤50 y, 95% CI 3.4%–4.8%) to 46.6% (≥80 y, 95% CI 45.2%–48.1%) by 5 y following an upper gastrointestinal bleed.

**Figure 6:**
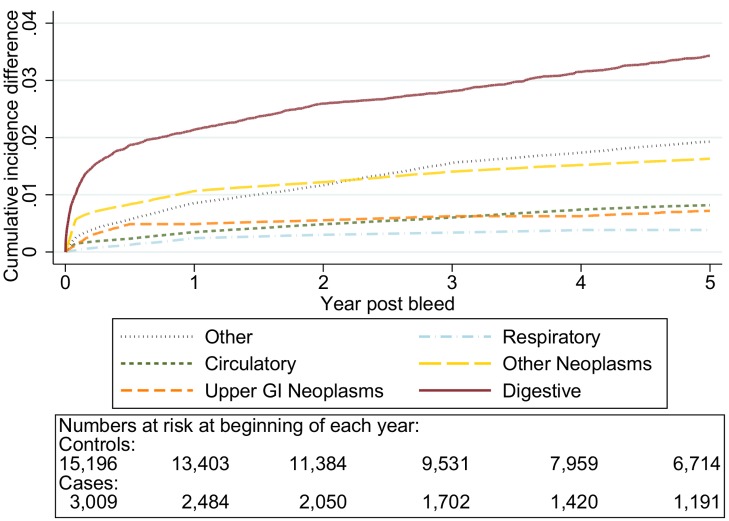
Excess cumulative incidence function for each cause of death following non-variceal bleeding ≤50 y. Excess death by ICD 10 chapter over 5 y follow-up following a bleed compared to age, sex, year, and practice matched controls. Numbers at risk at beginning of each year of follow-up shown beneath legend. Adjusted for gender and competing risks.

**Figure 7:**
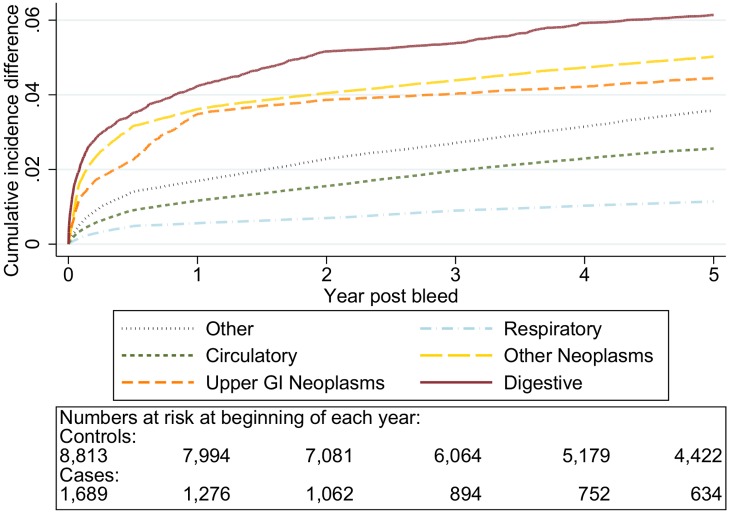
Excess cumulative incidence function for each cause of death following non-variceal bleeding 50–59 y. Excess death by ICD 10 chapter over 5 y follow-up following a bleed compared to age, sex, year, and practice matched controls. Numbers at risk at beginning of each year of follow-up shown beneath legend. Adjusted for gender and competing risks.

**Figure 8:**
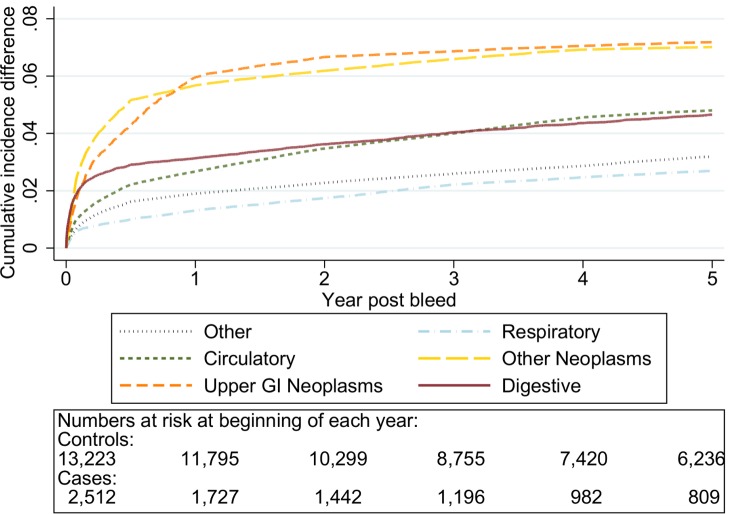
Excess cumulative incidence function for each cause of death following non-variceal bleeding 60–69 y. Excess death by ICD 10 chapter over 5 y follow-up following a bleed compared to age, sex, year, and practice matched controls. Numbers at risk at beginning of each year of follow-up shown beneath legend. Adjusted for gender and competing risks.

**Figure 9:**
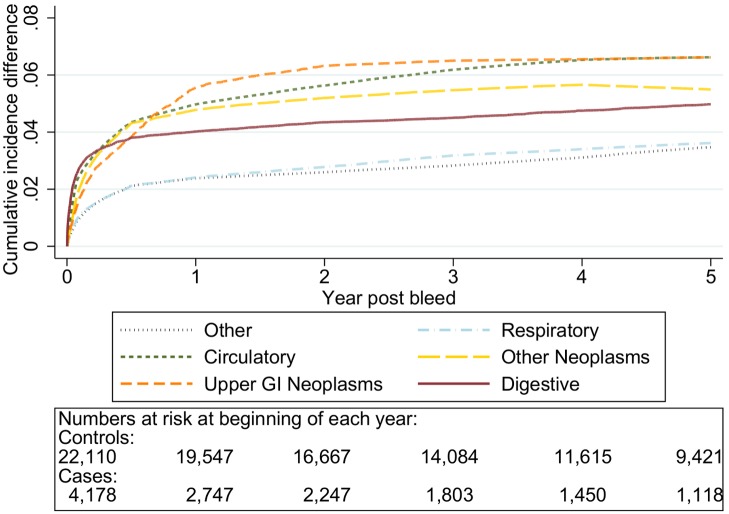
Excess cumulative incidence function for each cause of death following non-variceal bleeding 70–79 y. Excess death by ICD 10 chapter over 5 y follow-up following a bleed compared to age, sex, year, and practice matched controls. Numbers at risk at beginning of each year of follow-up shown beneath legend. Adjusted for gender and competing risks.

**Figure 10:**
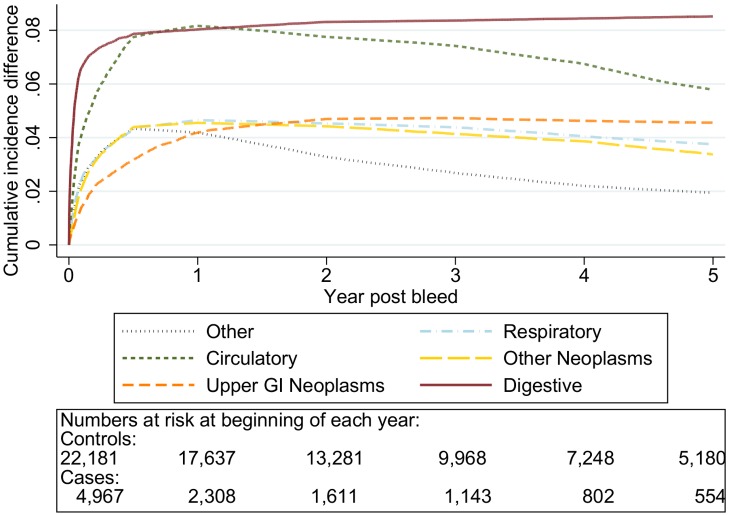
Cumulative incidence function following non-variceal bleeding for cause of death ≥80 y. Excess death by ICD 10 chapter over 5 y follow-up following a bleed compared to age, sex, year, and practice matched controls. Numbers at risk at beginning of each year of follow-up shown beneath legend. Adjusted for gender and competing risks.

The graphs in Figures 6–10 show the excess CIF associated with a bleed adjusted for competing risks. Overall there was an excess CIF of 26% compared to matched controls and this peaked in the 70–79-y-old age group. The excess CIF for death due to malignant or non-malignant gastrointestinal causes ranged from between 3.6% (≤50 y, 95% CI 3.0%–4.2%) to 13.4% (≥80 y, 95% CI 12.4%–14.5%). In contrast the excess CIF for death due to non-gastrointestinal causes ranged from 3.8% (≤50 y, 95% CI 3.1%–4.5%) to 19.0% (≥80 y, 95% CI 17.5%–20.6%). Therefore over half the excess CIF was due to non-gastrointestinal causes of death. Table 3 shows that the 95% CIs for the excess CIF values exclude the null for all causes of death apart from respiratory disease (which were not interpretable due to small numbers).

**Table 3:** Excess cumulative incidence function post bleed by time post bleed.

Cause of Death	Age Range	1 mo		1 y		5 y	
		eCIF	(95% CI)	eCIF	(95% CI)	eCIF	(95% CI)
**Upper GI Neoplasms**	≤50 y	0.11	(−0.00 to 0.22)	0.43	(0.22–0.64)	0.61	(0.33–0.89)
	50–59 y	1.15	(−0.30 to 2.59)	3.11	(1.53–4.68)	3.62	(2.02–5.22)
	60–69 y	1.76	(−0.35 to 3.87)	5.58	(3.33–7.82)	6.58	(4.28–8.89)
	70–79 y	1.47	(−0.28 to 3.21)	4.76	(2.91–6.61)	5.65	(3.70–7.59)
	≥80 y	1.20	(−0.12 to 2.52)	3.83	(2.35–5.31)	4.41	(2.79–6.03)
**Other Neoplasms**	≤50 y	0.51	(0.27–0.75)	1.00	(0.64–1.36)	1.29	(0.86–1.72)
**(Not Upper GI)**	50–59 y	1.45	(0.93–1.96)	3.45	(2.63–4.27)	4.17	(3.17–5.18)
	60–69 y	2.39	(1.83–2.96)	5.65	(4.54–6.75)	6.64	(5.22–8.06)
	70–79 y	1.82	(1.39–2.25)	4.68	(3.70–5.65)	5.23	(3.87–6.59)
	≥80 y	1.95	(1.50–2.40)	4.82	(3.52–6.11)	4.38	(2.65–6.12)
**Cardiovascular**	≤50 y	0.14	(0.02–0.26)	0.38	(0.17–0.60)	0.62	(0.32–0.92)
	50–59 y	0.37	(0.11–0.64)	1.44	(0.92–1.96)	2.36	(1.53–3.19)
	60–69 y	1.12	(0.74–1.49)	3.04	(2.31–3.78)	4.66	(3.52–5.80)
	70–79 y	2.40	(1.89–2.91)	5.25	(4.19–6.32)	6.44	(4.76–8.12)
	≥80 y	3.92	(3.21–4.63)	8.66	(6.30–11.03)	7.64	(3.97–11.30)
**Respiratory** a	≤50 y	0.03	—	0.24	—	0.31	—
	50–59 y	0.16	—	0.58	—	1.04	—
	60–69 y	0.56	—	1.28	—	2.08	—
	70–79 y	0.97	—	2.46	—	3.24	—
	≥80 y	2.28	—	4.95	—	4.56	—
**Digestive**	≤50 y	1.01	(0.28–1.75)	2.28	(1.43–3.14)	3.04	(2.06–4.02)
	50–59 y	1.96	(0.25–3.67)	4.04	(2.23–5.84)	5.33	(3.45–7.22)
	60–69 y	1.93	(0.27–3.59)	3.15	(1.47–4.83)	3.90	(2.17–5.63)
	70–79 y	2.60	(0.67–4.53)	4.01	(2.05–5.98)	4.45	(2.46–6.43)
	≥80 y	6.43	(2.43–10.42)	8.19	(4.20–12.17)	8.56	(4.58–12.54)
**Other**	≤50 y	0.26	(0.09–0.44)	0.95	(0.61–1.29)	1.65	(1.10–2.19)
	50–59 y	0.53	(0.21–0.85)	1.90	(1.26–2.53)	3.09	(2.17–4.02)
	60–69 y	0.70	(0.42–0.98)	1.85	(1.31–2.38)	2.37	(1.63–3.11)
	70–79 y	0.92	(0.65–1.19)	2.58	(1.99–3.17)	3.06	(2.15–3.97)
	≥80 y	2.29	(1.78–2.80)	4.37	(2.96–5.78)	2.54	(0.58–4.50)

**95% CIs obtained by bootstrapping (500 iterations).**

a CIs were too unstable to be interpretable for respiratory causes of death due to small numbers.

eCIF, excess CIF; the absolute difference in the CIF between patients with non-variceal bleeding and age, sex, year, and general practice matched controls without non-variceal bleeding; GI, gastrointestinal.

### Sensitivity Analyses

Table 4 shows the excess mortality at 5 y associated with a bleed when adjusted for prior co-morbidity, alcohol, or smoking. Adjusting for smoking and alcohol had no effect on the excess mortality, whilst adjusting for prior co-morbidity slightly reduced the point estimates for non-gastrointestinal co-morbidity. However the significant excess risk of death for all causes persisted, with CIs overlapping with those from the main analysis. When we examined in more detail the prior medical history of patients exposed to a bleed, 54% of those who subsequently died from a neoplasm did not have a neoplasm coded before the bleed, and 41% of those who died from a cardiovascular death did not have cardiovascular disease coded before the bleed. Finally when examined by bleed site the excess risks were unchanged from the main analysis (Table S4).

**Table 4:** Excess cumulative incidence function at 5 y post bleed by age group, adjusted for lifestyle factors and co-morbidity.

Causes of Death	Age Range	Adjusted for Gender only^a^		Adjusted for Alcohol, Smoking, and Gender		Adjusted for Co-morbidity, Alcohol, Smoking, and Gender	
		eCIF	(95% CI)	eCIF	(95% CI)	eCIF	(95% CI)
**Upper GI neoplasms**	≤50 y	0.61	(0.33–0.89)	0.63	(0.33–0.92)	0.62	(0.34–0.90)
	50–59 y	3.62	(2.02–5.22)	3.79	(2.55–5.03)	3.52	(2.30–4.74)
	60–69 y	6.58	(4.28–8.89)	6.67	(4.88–8.46)	6.00	(4.08–7.92)
	70–79 y	5.65	(3.70–7.59)	5.63	(4.21–7.05)	5.05	(3.51–6.58)
	≥80 y	4.41	(2.79–6.03)	4.39	(3.07–5.71)	4.08	(2.65–5.50)
**Other neoplasms (not upper GI)**	≤50 y	1.29	(0.86–1.72)	1.26	(0.78–1.75)	1.17	(0.74–1.60)
	50–59 y	4.17	(3.17–5.18)	4.05	(2.67–5.42)	3.20	(2.06–4.33)
	60–69 y	6.64	(5.22–8.06)	6.39	(4.38–8.39)	4.67	(2.94–6.40)
	70–79 y	5.23	(3.87–6.59)	5.11	(3.39–6.82)	3.64	(2.22–5.06)
	≥80 y	4.38	(2.65–6.12)	4.34	(2.4–6.28)	3.19	(1.62–4.76)
**Cardiovascular**	≤50 y	0.62	(0.32–0.92)	0.65	(0.32–0.97)	0.50	(0.25–0.74)
	50–59 y	2.36	(1.53–3.19)	2.21	(1.29–3.14)	1.49	(0.82–2.15)
	60–69 y	4.66	(3.52–5.80)	4.50	(2.95–6.04)	2.89	(1.75–4.02)
	70–79 y	6.44	(4.76–8.12)	6.32	(4.05–8.59)	4.02	(2.34–5.70)
	≥80 y	7.64	(3.97–11.30)	7.66	(3.41–11.91)	5.32	(2.16–8.48)
**Respiratory** b	≤50 y	0.31	—	0.26	—	0.20	—
	50–59 y	1.04	—	0.99	—	0.69	—
	60–69 y	2.08	—	1.92	—	1.28	—
	70–79 y	3.24	—	3.15	—	2.08	—
	≥80 y	4.56	—	4.51	—	3.21	—
**Digestive**	≤50 y	3.04	(2.06–4.02)	2.38	(1.7–3.05)	2.36	(1.75–2.97)
	50–59 y	5.33	(3.45–7.22)	4.20	(2.98–5.42)	4.05	(2.82–5.27)
	60–69 y	3.90	(2.17–5.63)	3.53	(2.51–4.55)	3.36	(2.25–4.47)
	70–79 y	4.45	(2.46–6.43)	4.09	(3.13–5.06)	3.88	(2.74–5.02)
	≥80 y	8.56	(4.58–12.54)	8.23	(6.33–10.13)	8.01	(5.82–10.20)
**Other**	≤50 y	1.65	(1.10–2.19)	1.55	(0.95–2.14)	1.39	(0.89–1.89)
	50–59 y	3.09	(2.17–4.02)	3.11	(2.01–4.21)	2.65	(1.68–3.64)
	60–69 y	2.37	(1.63–3.11)	2.46	(1.51–3.41)	2.06	(1.16–2.96)
	70–79 y	3.06	(2.15–3.97)	3.29	(2.09–4.48)	2.78	(1.64–3.93)
	≥80 y	2.54	(0.58–4.50)	2.73	(0.45–5.01)	2.72	(0.42–5.02)

**95% CIs obtained by bootstrapping (500 iterations).**

a As in Table 3.

b CIs were too unstable to be interpretable for respiratory causes of death due to small numbers.

eCIF, excess CIF; the absolute difference in the CIF between patients with non-variceal bleeding and age, sex, year, and general practice matched controls without non-variceal bleeding; GI, gastrointestinal.

## Discussion

We determined the cumulative excess risk of death in the 5 y following a non-variceal upper gastrointestinal bleed. We have done this in a large unselected population cohort and assessed the underlying cause whilst adjusting for competing risks. This analysis showed that although there was an excess risk of death from gastrointestinal causes, more than half the total excess risk of death was from unrelated non-gastrointestinal causes. The largest absolute increases were from neoplastic and cardiovascular disease, but half of those who died from these two causes were not diagnosed prior to the upper gastrointestinal bleed. This finding suggests that, in addition to indicating upper gastrointestinal pathology, an upper gastrointestinal bleed is either a cause of non-gastrointestinal co-morbidity or an indicator of existing co-morbidity (whether diagnosed or undiagnosed). Our findings contrast with those for other acute life-limiting medical events. For example, three-quarters of the excess death following a myocardial infarction were shown to be due to the cardiovascular disease, and two-thirds of the excess death following a stroke were shown to be due to related respiratory infections, cardiovascular disease, or the cerebrovascular disease itself [1],[2].

The main strengths of this study compared to previous studies are its larger size, follow-up, competing risk adjustment, and general population setting. These factors allowed us to calculate accurate, unbiased, and more detailed mortality rates for different causes of death than has previously been done to our knowledge. We used linked electronic primary and secondary health care records in which the definition of bleeding was previously found to be accurate. In HES the incidence of peptic ulcer haemorrhage (1992–1995) was comparable to the 1993 regional BSG audit (32 versus 29 per 100,000 per year, respectively) [18]. More recently similar numbers of all upper gastrointestinal bleed hospital admissions and related procedures were recorded in HES as were recorded in the 2007 prospective national UK audit [19]. In the GPRD the positive predictive value of an upper gastrointestinal bleed code was 99% using anonymised chart review [20],[21]. We further strengthened the case definition for our study by requiring evidence from both databases to be present to define a bleed [13].

A possible weakness of our study is the potentially imperfect data on some recognised risk factors for the excess mortality. These data may have caused us to overestimate the excess mortality associated with upper gastrointestinal bleeding. However, the GPRD contains comprehensive recording of all available diagnoses and prescriptions, and with the addition of information from hospital records, any bias from underreporting of co-morbidities will have been minimised. Underreporting might have occurred for alcohol and smoking; however, these risk factors did not show any important confounding of the association between upper gastrointestinal bleeding and excess mortality at their current level of ascertainment, so it is unlikely any increased reporting would account for the magnitude of the association we identified.

The information on the fact and cause of death in our study is from the Office of National Statistics death registry, which uses standardised WHO guidelines to extract information from death certificates. Although death certificates can sometimes be imprecise, they are the official legal requirement for ascertaining the cause of death. As a consequence, death certificates are the only standardised method to extract cause of death information across a large population. Here, the underlying cause of death information was used to avoid the effect of changes in coding requirements over time [22].

Although the Charlson index was not directly developed for routine records, longer term follow-up, or cause specific mortality, we believe its original purpose in predicting all cause mortality using diagnosed co-morbidity was suitably similar to our study to be useful [17]. Furthermore the Charlson index has been validated in many different contexts including routine primary care and hospital admission data [23]–[26]. Other co-morbidity scores that could be used, such as the Elixhauser index or a simple counts of diagnoses, have been validated less frequently and in fewer contexts. However, some of these other scores also include other outcomes, such as financial cost, which are not necessarily a measure of the predicted mortality from a disease. The Charlson index was therefore selected as the most appropriate co-morbidity score for our study.

Previous studies of cause of death following upper gastrointestinal bleeding were identified by a PubMed search (using the search terms “Cause of death”[Mesh] AND (“Peptic Ulcer”[Mesh] OR “Gastrointestinal hemorrhage”[Mesh]) across all years up to September 2012) and by examining the references of selected papers from the search results. There were a number of studies of cause of death during the first 30 d following an upper gastrointestinal bleed [5],[6],[27]–[29]. The largest was from Hong Kong; however, it assessed only peptic ulcer bleeds from one hospital. Furthermore it only reported deaths from each cause with no comparison group [6].

In contrast, there have been only a few studies examining causes of death in the long term following a bleed. Studies in the 1980s and 1990s followed up peptic ulcer cohorts post surgical treatment rather than upper gastrointestinal bleeds (shown in Table 5). These studies were susceptible to the selection bias inherent in surgical cohorts [30], and furthermore they are now dated as the cohorts were completed in the 1980s before ulcer treatment was radically changed by the introduction of *Helicobacter pylori* eradication [31] and proton pump inhibitors [32],[33]. The studies that did follow up upper gastrointestinal bleeding included only patients with proven peptic ulcers who had survived the first 30 d (Table 5). The largest study by Ruigomez et al. consisted of 978 patients with 155 deaths [4]. However, cause of death information was not available and cause of death was imputed by the most recently recorded co-morbidity, increasing the risk of misclassification. An upper age limit also meant that the study's age distribution differed considerably from our unselected cohort, so it was no longer representative of those currently presenting with bleeds. The next largest and arguably better study was able to obtain death certificate data from the national death register and was therefore similar to our study in being able to ascertain the causes of death in a standardised manner. However, the study was restricted to one city and to patients over 60 y old who were hospitalised with endoscopically proven peptic ulcers (*n = *487, deaths = 142). This methodology limits the study's generalisability to a contemporary population and introduces a selection bias towards those deemed suitable for an endoscopy [3]. In both studies mortality rates were not calculated, no adjustment for competing risks was made, and neither study had the power to assess causes of death by age or time post bleed. In contrast, we have been able to calculate stratified excess risks for different causes of death adjusted for competing risks within a large population-based cohort.

**Table 5:** Previous literature on long term outcome following peptic ulcer cohorts with follow-up over 30 d.

First Author	Operation	Year Published [citation]	Follow-up (y)	Total Deaths	Cause of Death (Percent of All Deaths)					
					Neoplasms			Cardiovascular^a^	Respiratory	Digestive^b^
					Any	Upper GI	Respiratory			
**Caygill**	Vagotomy	1991 [34]	?	577	32.8	2.6	12.3	—	—	—
**McIntosh**	Gastric ulcer cohort	1991 [35]	10	305	17.4	1.6	4.6	51.5	10.8	7.2
**Macintyre**	Duodenal ulcer operation	1994 [36]	<20	791	31.7	0.02	0.03	35.5	8.0	2.8
**Lindell**	Unoperated peptic ulcer	1994 [37]	12	121	26.4	5.0	—	47.1	—	9.9
**Staël von Holstein**	Partial gastrectomy	1995 [30]	<20	399	22.3	1.8	7.5	49.9	9.5	5.3
**Svanes**	Perforated peptic ulcer	1999 [38]	18.8	817	10.8	1.2	3.8	13.8	4.0	8.8
**Duggan**	Peptic ulcer operation	1999 [39]	<20	224	10.7	—	—	42.9	13.8	10.3
**Smart**	Bleeding ulcer cohort	1986 [9]	<8	77	16.9	1.3	1.3	24.7	5.2	6.5
**Rorbaek-Madsen**	Bleeding ulcer cohort	1994 [7]	<8	45	—	—	—	—	—	6.7
**Kubba**	Bleeding ulcer cohort	1997 [8]	<6.5	30	10.0	—	—	66.7	16.7	6.7
**Hudson**	Bleeding ulcer cohort	1995 [3]	2.8 (mean)	142	23.9	3.5	7.0	34.5	19.7	5.6
**Ruigomez**	Bleeding ulcer cohort	2000 [4]	2.8 (mean)	155	12.9	—	—	36.8	17.4	9.0

We searched PubMed with no restrictions for “Cause of death”[Mesh] AND (“Peptic Ulcer”[Mesh] OR “Gastrointestinal hemorrhage”[Mesh]) and also examined the references of selected papers from the search results.

a Cardiovascular definitions varied from ischaemic heart disease only to including cerebrovascular disease.

b Digestive disease definitions varied from peptic ulcer related to non-malignant GI disease.

GI, gastrointestinal.

We found a considerable excess of all causes of death in individuals following a non-variceal upper gastrointestinal bleed, and over half of these deaths were due to non-gastrointestinal co-morbidity, particularly neoplastic and cardiovascular disease. This excess in deaths was not explained by co-morbidity such as cancer or cardiovascular disease diagnosed prior to the admission. Therefore, an upper gastrointestinal bleed may be a marker of disease or an indicator of a deterioration in non-gastrointestinal co-morbidity. Consequently, this analysis suggests that for patients who have a non-variceal upper gastrointestinal bleed, re-assessment of co-morbidity should be considered in the follow-up period.

## Supporting Information

Table S1 **Mortality rate per 100 person-years in patients with no upper gastrointestinal bleeding, stratified by cause of death by ICD 10 headings in the 5 y post matching to a bleed case.**
(DOC)

Table S2 **Mortality rate per 100 person-years in patients following an upper gastrointestinal bleed, stratified by cause of death by ICD 10 headings and age group in the 5 y post bleed.**
(DOC)

Table S3 **Mortality rate per 100 person-years in patients with no upper gastrointestinal bleeding, stratified by cause of death by ICD 10 headings and age group in the 5 y post matching to a bleed case.**
(DOC)

Table S4 **Excess cumulative incidence function 5 y post upper gastrointestinal bleed by age group and recorded site of bleeding. 95% CIs obtained by bootstrapping (500 iterations).**
(DOC)

## Abbreviations

CIF: cumulative incidence function
GPRD: General Practice Research Database
HES: Hospital Episodes Statistics
ICD 10: International Classification of Diseases version 2010
