# Supplementary material for: Excess Long-Term Mortality following Non-Variceal Upper Gastrointestinal Bleeding: A Population-Based Cohort Study
Source: PLoS Med. 2013 Apr 30;10(4):e1001437. doi: 10.1371/journal.pmed.1001437 (PMC3640094; doi:10.1371/journal.pmed.1001437)
Supplement: Table S1 — Mortality rate per 100 person-years in patients with no upper gastrointestinal bleeding, stratified by cause of death by ICD 10 headings in the 5 y post matching to a bleed case. (DOC) [file pmed.1001437.s001.doc]

Table S1: **Mortality rate per 100 person years in patients with no upper gastrointestinal bleeding, stratified by cause of death by ICD10 headings in the 5 years post matching to a bleed case.**

|  | **1st month deaths (n)** | **Rate** | **95% CI** | **1 month to 1 year deaths (n)** | **Rate** | **95% CI** | **1 year to 5 years deaths (n)** | **Rate** | **95% CI** |
| --- | --- | --- | --- | --- | --- | --- | --- | --- | --- |
| **Neoplasms** | **65** | **1.0** | **(0.8-1.2)** | **684** | **1.0** | **(0.9-1.1)** | **1866** | **0.9** | **(0.9-1.0)** |
| Cancer- Oesophagus | ≤5 |  |  | 31 | 0.0 | (0.0-0.1) | 81 | 0.0 | (0.0-0.1) |
| Cancer- Stomach | ≤5 |  |  | 21 | 0.0 | (0.0-0.0) | 61 | 0.0 | (0.0-0.0) |
| Cancer- Colon | 7 | 0.1 | (0.0-0.2) | 33 | 0.0 | (0.0-0.1) | 112 | 0.1 | (0.0-0.1) |
| Cancer- Pancreas | ≤5 |  |  | 26 | 0.0 | (0.0-0.1) | 92 | 0.0 | (0.0-0.1) |
| Cancer- Respiratory | 10 | 0.1 | (0.1-0.3) | 140 | 0.2 | (0.2-0.2) | 397 | 0.2 | (0.2-0.2) |
| Cancer- Skin or Bone | ≤5 |  |  | 27 | 0.0 | (0.0-0.1) | 70 | 0.0 | (0.0-0.0) |
| Cancer- Breast | ≤5 |  |  | 48 | 0.1 | (0.1-0.1) | 93 | 0.0 | (0.0-0.1) |
| Cancer- Prostate | 7 | 0.1 | (0.0-0.2) | 83 | 0.1 | (0.1-0.1) | 190 | 0.1 | (0.1-0.1) |
| **Circulatory** | **128** | **1.9** | **(1.6-2.2)** | **1089** | **1.6** | **(1.5-1.7)** | **3226** | **1.6** | **(1.6-1.7)** |
| Rheumatic disease | ≤5 |  |  | ≤5 |  |  | 15 | 0.0 | (0.0-0.0) |
| Hypertensive disease | ≤5 |  |  | 18 | 0.0 | (0.0-0.0) | 62 | 0.0 | (0.0-0.0) |
| IHD | 46 | 0.7 | (0.5-0.9) | 391 | 0.6 | (0.5-0.6) | 1414 | 0.7 | (0.7-0.8) |
| Pulmonary circulatory disease | ≤5 |  |  | 17 | 0.0 | (0.0-0.0) | 50 | 0.0 | (0.0-0.0) |
| Heart - other | 13 | 0.2 | (0.1-0.3) | 130 | 0.2 | (0.2-0.2) | 365 | 0.2 | (0.2-0.2) |
| CVA | 43 | 0.6 | (0.5-0.9) | 352 | 0.5 | (0.5-0.6) | 959 | 0.5 | (0.5-0.5) |
| **Respiratory** | **46** | **0.7** | **(0.5-0.9)** | **441** | **0.6** | **(0.6-0.7)** | **1237** | **0.6** | **(0.6-0.7)** |
| Respiratory infections | 16 | 0.2 | (0.1-0.4) | 174 | 0.2 | (0.2-0.3) | 558 | 0.3 | (0.3-0.3) |
| Chronic Airway disease | 14 | 0.2 | (0.1-0.3) | 124 | 0.2 | (0.1-0.2) | 389 | 0.2 | (0.2-0.2) |
| ILD | ≤5 |  |  | 28 | 0.0 | (0.0-0.1) | 92 | 0.0 | (0.0-0.1) |
| **Digestive** | **≤5** |  |  | **105** | **0.2** | **(0.1-0.2)** | **281** | **0.1** | **(0.1-0.2)** |
| Upper GI | ≤5 |  |  | 31 | 0.0 | (0.0-0.1) | 55 | 0.0 | (0.0-0.0) |
| Lower GI | ≤5 |  |  | 49 | 0.1 | (0.1-0.1) | 158 | 0.1 | (0.1-0.1) |
| Liver or gallbladder | ≤5 |  |  | 22 | 0.0 | (0.0-0.0) | 54 | 0.0 | (0.0-0.0) |
| Pancreas | ≤5 |  |  | ≤5 |  |  | 12 | 0.0 | (0.0-0.0) |
| **Other** | **63.0** | **0.9** | **(0.7-1.2)** | **515** | **0.7** | **(0.7-0.8)** | **1431** | **0.7** | **(0.7-0.8)** |
| **Uncoded** | **20.0** | **0.3** | **(0.2-0.5)** | **155** | **0.2** | **(0.2-0.3)** | **287** | **0.1** | **(0.1-0.2)** |
| **Total** | **326.0** | **4.8** | **(4.3-5.4)** | **2989** | **4.3** | **(4.1-4.4)** | **8328** | **4.2** | **(4.1-4.3)** |

**Bold** headings indicate ICD 10 chapter headings and non bold headings indicate ICD 10  subchapter headings

Due to anonymisation numbers in cells with 5 or less events are not shown and “Other” subchapters under each heading are not shown.
