# Supplementary material for: Excess Long-Term Mortality following Non-Variceal Upper Gastrointestinal Bleeding: A Population-Based Cohort Study
Source: PLoS Med. 2013 Apr 30;10(4):e1001437. doi: 10.1371/journal.pmed.1001437 (PMC3640094; doi:10.1371/journal.pmed.1001437)
Supplement: Table S2 — Mortality rate per 100 person-years in patients following an upper gastrointestinal bleed, stratified by cause of death by ICD 10 headings and age group in the 5 y post bleed. (DOC) [file pmed.1001437.s002.doc]

Table S2: **Mortality rate per 100 person years in patients following an upper gastrointestinal bleed, stratified by cause of death by ICD10 headings and age group in the 5 years post bleed.**

|  | **≤60 yrs deaths (n)** | **Rate** | **95% CI** | **60-69 yrs deaths (n)** | **Rate** | **95% CI** | **70-79 yrs deaths (n)** | **Rate** | **95% CI** | **≥80 yrs deaths (n)** | **Rate** | **95% CI** |
| --- | --- | --- | --- | --- | --- | --- | --- | --- | --- | --- | --- | --- |
| **Neoplasms** | **158** | **1.1** | (0.9-1.3) | **296** | **4.5** | (4.0-5.0) | **487** | **4.8** | (4.4-5.2) | **486** | **6.4** | (5.9-7.0) |
| Oesophagus | 21 | 0.1 | (0.1-0.2) | 55 | 0.8 | (0.6-1.1) | 73 | 0.7 | (0.6-0.9) | 55 | 0.7 | (0.6-0.9) |
| Stomach | 20 | 0.1 | (0.1-0.2) | 37 | 0.6 | (0.4-0.8) | 74 | 0.7 | (0.6-0.9) | 73 | 1.0 | (0.8-1.2) |
| Colon | 7 | 0.0 | (0.0-0.1) | 9 | 0.1 | (0.1-0.3) | 23 | 0.2 | (0.1-0.3) | 33 | 0.4 | (0.3-0.6) |
| Pancreas | 9 | 0.1 | (0.0-0.1) | 27 | 0.4 | (0.3-0.6) | 26 | 0.3 | (0.2-0.4) | 23 | 0.3 | (0.2-0.5) |
| Digestive(other) | 17 | 0.1 | (0.1-0.2) | 21 | 0.3 | (0.2-0.5) | 32 | 0.3 | (0.2-0.4) | 34 | 0.4 | (0.3-0.6) |
| Respiratory | 16 | 0.1 | (0.1-0.2) | 37 | 0.6 | (0.4-0.8) | 65 | 0.6 | (0.5-0.8) | 45 | 0.6 | (0.4-0.8) |
| Skin or Bone | ≤5 |  |  | ≤5 |  |  | 10 | 0.1 | (0.1-0.2) | 8 | 0.1 | (0.1-0.2) |
| Breast | 8 | 0.1 | (0.0-0.1) | 13 | 0.2 | (0.1-0.3) | 14 | 0.1 | (0.1-0.2) | 13 | 0.2 | (0.1-0.3) |
| Prostate | ≤5 |  |  | 18 | 0.3 | (0.2-0.4) | 29 | 0.3 | (0.2-0.4) | 48 | 0.6 | (0.5-0.8) |
| **Circulatory** | **68** | **0.5** | (0.4-0.6) | **135** | **2.0** | (1.7-2.4) | **388** | **3.8** | (3.4-4.2) | **735** | **9.7** | (9.0-10.4) |
| Rheumatic disease | ≤5 |  |  | ≤5 |  |  | 10 | 0.1 | (0.1-0.2) | 8 | 0.1 | (0.1-0.2) |
| Hypertensive disease | ≤5 |  |  | ≤5 |  |  | 8 | 0.1 | (0.0-0.2) | 10 | 0.1 | (0.1-0.2) |
| IHD | 33 | 0.2 | (0.2-0.3) | 58 | 0.9 | (0.7-1.1) | 165 | 1.6 | (1.4-1.9) | 245 | 3.2 | (2.9-3.7) |
| Pulmonary circulatory disease | ≤5 |  |  | ≤5 |  |  | 10 | 0.1 | (0.1-0.2) | 14 | 0.2 | (0.1-0.3) |
| Heart - other | 13 | 0.1 | (0.1-0.2) | 21 | 0.3 | (0.2-0.5) | 46 | 0.4 | (0.3-0.6) | 137 | 1.8 | (1.5-2.1) |
| CVA | 12 | 0.1 | (0.0-0.1) | 30 | 0.5 | (0.3-0.6) | 99 | 1.0 | (0.8-1.2) | 253 | 3.3 | (3.0-3.8) |
| **Respiratory** | **28** | **0.2** | (0.1-0.3) | **54** | **0.8** | (0.6-1.1) | **176** | **1.7** | (1.5-2.0) | **340** | **4.5** | (4.0-5.0) |
| Respiratory infections | 11 | 0.1 | (0.0-0.1) | 10 | 0.2 | (0.1-0.3) | 57 | 0.6 | (0.4-0.7) | 171 | 2.3 | (1.9-2.6) |
| Chronic Airway disease | 10 | 0.1 | (0.0-0.1) | 31 | 0.5 | (0.3-0.7) | 76 | 0.7 | (0.6-0.9) | 62 | 0.8 | (0.6-1.0) |
| ILD | ≤5 |  |  | ≤5 |  |  | 15 | 0.1 | (0.1-0.2) | 25 | 0.3 | (0.2-0.5) |
| **Digestive** | **120** | **0.8** | (0.7-1.0) | **59** | **0.9** | (0.7-1.1) | **106** | **1.0** | (0.9-1.3) | **149** | **2.0** | (1.7-2.3) |
| Upper GI | 11 | 0.1 | (0.0-0.1) | 8 | 0.1 | (0.1-0.2) | 43 | 0.4 | (0.3-0.6) | 77 | 1.0 | (0.8-1.3) |
| Lower GI | 11 | 0.1 | (0.0-0.1) | 9 | 0.1 | (0.1-0.3) | 34 | 0.3 | (0.2-0.5) | 49 | 0.6 | (0.5-0.9) |
| Liver or gallbladder | 95 | 0.7 | (0.5-0.8) | 36 | 0.5 | (0.4-0.8) | 24 | 0.2 | (0.2-0.3) | 19 | 0.3 | (0.2-0.4) |
| Pancreas | ≤5 |  |  | 6 | 0.1 | (0.0-0.2) | ≤5 |  |  | ≤5 |  |  |
| **Other** | **91** | **0.6** | (0.5-0.8) | **53** | **0.8** | (0.6-1.0) | **181** | **1.8** | (1.5-2.0) | **343** | **4.5** | (4.1-5.0) |
| **Uncoded** | **7** | **0.0** | (0.0-0.1) | **13** | **0.2** | (0.1-0.3) | **9** | **0.1** | (0.0-0.2) | **30** | **0.4** | (0.3-0.6) |
| **Total** | **472** | **3.3** | (3.0-3.6) | **610** | **9.2** | (8.5-9.9) | **1347** | **13.2** | (12.5-13.9) | **2083** | **27.5** | (26.3-28.7) |

1st year excluded

**Bold** headings indicate ICD 10 chapter headings and non bold headings indicate ICD 10  subchapter headings

Due to anonymisation numbers in cells with 5 or less events are not shown and “Other” subchapters under each heading are not shown.
