# Supplementary material for: Excess Long-Term Mortality following Non-Variceal Upper Gastrointestinal Bleeding: A Population-Based Cohort Study
Source: PLoS Med. 2013 Apr 30;10(4):e1001437. doi: 10.1371/journal.pmed.1001437 (PMC3640094; doi:10.1371/journal.pmed.1001437)
Supplement: Table S3 — Mortality rate per 100 person-years in patients with no upper gastrointestinal bleeding, stratified by cause of death by ICD 10 headings and age group in the 5 y post matching to a bleed case. (DOC) [file pmed.1001437.s003.doc]

**Table S3: Mortality rate per 100 person years in patients with no upper gastrointestinal bleeding, stratified by cause of death by ICD10 headings and age group in the 5 years post matching to a bleed case.**

|  | **≤60 yrs deaths (n)** | **Rate** | **95% CI** | **60-69 yrs deaths (n)** | **Rate** | **95% CI** | **70-79 yrs deaths (n)** | **Rate** | **95% CI** | **≥80 yrs old deaths (n)** | **Rate** | **95% CI** |
| --- | --- | --- | --- | --- | --- | --- | --- | --- | --- | --- | --- | --- |
| **Neoplasms** | **105** | **0.1** | **(0.1-0.2)** | **307** | **0.7** | **(0.6-0.7)** | **980** | **1.3** | **(1.2-1.4)** | **1158** | **1.9** | **(1.8-2.0)** |
| Cancer- Oesophagus | 6 | 0.0 | (0.0-0.0) | 19 | 0.0 | (0.0-0.1) | 45 | 0.1 | (0.0-0.1) | 42 | 0.1 | (0.1-0.1) |
| Cancer- Stomach | ≤5 |  |  | 7 | 0.0 | (0.0-0.0) | 37 | 0.0 | (0.0-0.1) | 35 | 0.1 | (0.0-0.1) |
| Cancer- Colon | ≤5 |  |  | 13 | 0.0 | (0.0-0.0) | 55 | 0.1 | (0.1-0.1) | 73 | 0.1 | (0.1-0.2) |
| Cancer- Pancreas | ≤5 |  |  | 14 | 0.0 | (0.0-0.1) | 44 | 0.1 | (0.0-0.1) | 55 | 0.1 | (0.1-0.1) |
| Cancer- Respiratory | 24 | 0.0 | (0.0-0.0) | 86 | 0.2 | (0.1-0.2) | 227 | 0.3 | (0.3-0.3) | 200 | 0.3 | (0.3-0.4) |
| Cancer- Skin or Bone | ≤5 |  |  | 16 | 0.0 | (0.0-0.1) | 38 | 0.0 | (0.0-0.1) | 38 | 0.1 | (0.0-0.1) |
| Cancer- Breast | 8 | 0.0 | (0.0-0.0) | 13 | 0.0 | (0.0-0.0) | 45 | 0.1 | (0.0-0.1) | 75 | 0.1 | (0.1-0.2) |
| Cancer- Prostate | 7 | 0.0 | (0.0-0.0) | 21 | 0.0 | (0.0-0.1) | 116 | 0.2 | (0.1-0.2) | 129 | 0.2 | (0.2-0.3) |
| **Circulatory** | **70** | **0.1** | **(0.1-0.1)** | **235** | **0.5** | **(0.4-0.6)** | **1182** | **1.6** | **(1.5-1.6)** | **2828** | **4.7** | **(4.5-4.9)** |
| Rheumatic disease | ≤5 |  |  | ≤5 |  |  | 10 | 0.0 | (0.0-0.0) | 6 | 0.0 | (0.0-0.0) |
| Hypertensive disease | ≤5 |  |  | ≤5 |  |  | 23 | 0.0 | (0.0-0.0) | 55 | 0.1 | (0.1-0.1) |
| IHD | 43 | 0.1 | (0.0-0.1) | 127 | 0.3 | (0.2-0.3) | 550 | 0.7 | (0.7-0.8) | 1085 | 1.8 | (1.7-1.9) |
| Pulmonary circulatory disease | ≤5 |  |  | 6 | 0.0 | (0.0-0.0) | 19 | 0.0 | (0.0-0.0) | 40 | 0.1 | (0.0-0.1) |
| Heart - other | ≤5 |  |  | 16 | 0.0 | (0.0-0.1) | 114 | 0.1 | (0.1-0.2) | 361 | 0.6 | (0.5-0.7) |
| CVA | 12 | 0.0 | (0.0-0.0) | 41 | 0.1 | (0.1-0.1) | 299 | 0.4 | (0.4-0.4) | 959 | 1.6 | (1.5-1.7) |
| **Respiratory** | **12** | **0.0** | **(0.0-0.0)** | **60** | **0.1** | **(0.1-0.2)** | **435** | **0.6** | **(0.5-0.6)** | **1171** | **2.0** | **(1.8-2.1)** |
| Respiratory infections | ≤5 |  |  | 8 | 0.0 | (0.0-0.0) | 125 | 0.2 | (0.1-0.2) | 596 | 1.0 | (0.9-1.1) |
| Chronic Airway disease | ≤5 |  |  | 38 | 0.1 | (0.1-0.1) | 194 | 0.3 | (0.2-0.3) | 278 | 0.5 | (0.4-0.5) |
| ILD | ≤5 |  |  | ≤5 |  |  | 41 | 0.1 | (0.0-0.1) | 70 | 0.1 | (0.1-0.1) |
| **Digestive** | **14** | **0.0** | **(0.0-0.0)** | **27** | **0.1** | **(0.0-0.1)** | **121** | **0.2** | **(0.1-0.2)** | **224** | **0.4** | **(0.3-0.4)** |
| Upper GI | ≤5 |  |  | ≤5 |  |  | 36 | 0.0 | (0.0-0.1) | 44 | 0.1 | (0.1-0.1) |
| Lower GI | ≤5 |  |  | 10 | 0.0 | (0.0-0.0) | 55 | 0.1 | (0.1-0.1) | 141 | 0.2 | (0.2-0.3) |
| Liver or gallbladder | 11 | 0.0 | (0.0-0.0) | 10 | 0.0 | (0.0-0.0) | 24 | 0.0 | (0.0-0.0) | 31 | 0.1 | (0.0-0.1) |
| Pancreas | ≤5 |  |  | ≤5 |  |  | 6 | 0.0 | (0.0-0.0) | 6 | 0.0 | (0.0-0.0) |
| **Other** | **44** | **0.1** | **(0.0-0.1)** | **62** | **0.1** | **(0.1-0.2)** | **365** | **0.5** | **(0.4-0.5)** | **1475** | **2.5** | **(2.3-2.6)** |
| **Uncoded** | **12** | **0.0** | **(0.0-0.0)** | **51** | **0.1** | **(0.1-0.1)** | **130** | **0.2** | **(0.1-0.2)** | **249** | **0.4** | **(0.4-0.5)** |
| **Total** | **257** | **0.3** | **(0.3-0.3)** | **742** | **1.6** | **(1.5-1.7)** | **3213** | **4.2** | **(4.1-4.4)** | **7105** | **11.9** | **(11.6-12.1)** |

1st year excluded

**Bold** headings indicate ICD 10 chapter headings and non bold headings indicate ICD 10  subchapter headings

Due to anonymisation numbers in cells with 5 or less events are not shown and “Other” subchapters under each heading are not shown.
