# Supplementary material for: Excess Long-Term Mortality following Non-Variceal Upper Gastrointestinal Bleeding: A Population-Based Cohort Study
Source: PLoS Med. 2013 Apr 30;10(4):e1001437. doi: 10.1371/journal.pmed.1001437 (PMC3640094; doi:10.1371/journal.pmed.1001437)
Supplement: Table S4 — Excess cumulative incidence function 5 y post upper gastrointestinal bleed by age group and recorded site of bleeding. 95% CIs obtained by bootstrapping (500 iterations). (DOC) [file pmed.1001437.s004.doc]

Table S4: **Excess cumulative incidence function 5 years post upper gastrointestinal bleed by age group and recorded site of bleeding. 95% confidence intervals obtained by bootstrapping (500 iterations).**

|  | **Oesophageal** | | **Gastric** | | **Duodenal** | | **Other or not specified** | |
| --- | --- | --- | --- | --- | --- | --- | --- | --- |
|  | **eCIF** | **95% CI** | **eCIF** | **95% CI** | **eCIF** | **95% CI** | **eCIF** | **95% CI** |
| **Upper GI Neoplasms** ≤ 50 years | 0.71 | (0.34-1.07) | 0.78 | (0.28-1.27) | 0.64 | (0.21-1.07) | 1.30 | (0.44-2.16) |
| 50-59 years | 4.58 | (2.45-6.70) | 4.96 | (2.02-7.89) | 4.08 | (1.58-6.58) | 8.04 | (3.40-12.68) |
| 60-69 years | 7.85 | (4.22-11.47) | 8.37 | (3.54-13.21) | 6.91 | (2.68-11.14) | 13.24 | (6.04-20.45) |
| 70-79 years | 7.18 | (3.65-10.71) | 7.65 | (2.72-12.58) | 6.18 | (1.93-10.43) | 12.10 | (5.09-19.12) |
| ≥ 80 years | 5.38 | (2.57-8.18) | 5.30 | (1.58-9.02) | 4.12 | (1.04-7.21) | 8.19 | (3.05-13.34) |
| **Other Neoplasms** ≤ 50 years | 1.46 | (0.80-2.11) | 1.55 | (0.83-2.27) | 1.46 | (0.73-2.19) | 2.36 | (1.18-3.54) |
| 50-59 years | 4.93 | (2.53-7.33) | 5.22 | (2.62-7.81) | 4.91 | (2.34-7.49) | 7.62 | (3.80-11.43) |
| 60-69 years | 6.93 | (3.07-10.80) | 7.30 | (3.15-11.46) | 6.86 | (2.77-10.95) | 10.55 | (5.10-15.99) |
| 70-79 years | 5.59 | (1.69-9.49) | 5.90 | (1.48-10.31) | 5.46 | (1.09-9.82) | 9.09 | (3.53-14.66) |
| ≥ 80 years | 4.21 | (0.27-8.15) | 4.21 | (0.04-8.37) | 3.54 | (-0.42-7.49) | 6.15 | (1.21-11.10) |
| **Cardiovascular** ≤ 50 years | 0.61 | (0.24-0.98) | 0.77 | (0.31-1.23) | 0.75 | (0.29-1.20) | 1.09 | (0.44-1.75) |
| 50-59 years | 2.04 | (0.71-3.38) | 2.65 | (1.06-4.24) | 2.55 | (0.96-4.15) | 3.62 | (1.49-5.75) |
| 60-69 years | 4.12 | (1.54-6.71) | 5.38 | (2.27-8.48) | 5.23 | (2.15-8.31) | 6.99 | (3.32-10.65) |
| 70-79 years | 4.45 | (0.12-8.79) | 6.79 | (1.47-12.11) | 6.53 | (1.22-11.83) | 9.06 | (3.02-15.10) |
| ≥ 80 years | 2.72 | (-4.92-10.36) | 5.64 | (-3.41-14.70) | 4.73 | (-4.15-13.61) | 7.16 | (-2.28-16.60) |
| **Respiratory***  ≤ 50 years | 0.36 | - | 0.36 | - | 0.39 | - | 0.56 | - |
| 50-59 years | 1.00 | - | 1.00 | - | 1.10 | - | 1.53 | - |
| 60-69 years | 2.37 | - | 2.34 | - | 2.61 | - | 3.46 | - |
| 70-79 years | 3.07 | - | 2.90 | - | 3.46 | - | 4.83 | - |
| ≥ 80 years | 3.27 | - | 3.35 | - | 3.76 | - | 5.46 | - |
| **Digestive** ≤ 50 years | 1.66 | (0.95-2.36) | 1.63 | (0.58-2.67) | 2.27 | (0.83-3.70) | 2.51 | (1.00-4.02) |
| 50-59 years | 2.98 | (1.56-4.41) | 2.94 | (0.96-4.91) | 4.09 | (1.35-6.84) | 4.35 | (1.58-7.11) |
| 60-69 years | 2.12 | (0.96-3.27) | 2.17 | (0.56-3.79) | 3.06 | (0.84-5.28) | 3.14 | (1.02-5.27) |
| 70-79 years | 2.03 | (0.73-3.33) | 1.98 | (0.03-3.93) | 3.10 | (0.43-5.77) | 3.10 | (0.69-5.52) |
| ≥ 80 years | 3.75 | (1.43-6.06) | 4.17 | (0.99-7.36) | 5.87 | (1.50-10.25) | 5.61 | (1.89-9.33) |
| **Other** ≤ 50 years | 1.92 | (1.02-2.82) | 1.76 | (0.90-2.63) | 1.83 | (0.94-2.71) | 2.71 | (1.37-4.05) |
| 50-59 years | 3.90 | (1.84-5.95) | 3.56 | (1.62-5.49) | 3.70 | (1.71-5.69) | 5.12 | (2.41-7.82) |
| 60-69 years | 2.98 | (1.07-4.89) | 2.72 | (0.97-4.47) | 2.90 | (1.08-4.72) | 3.92 | (1.70-6.13) |
| 70-79 years | 3.33 | (0.63-6.03) | 2.67 | (0.04-0.30) | 2.94 | (0.21-5.67) | 4.34 | (1.11-7.57) |
| ≥ 80 years | 1.19 | (-4.10-6.48) | 1.13 | (-3.62-5.87) | 1.61 | (-3.28-6.50) | 2.88 | (-2.50-8.26) |

eCIF - The absolute difference in the cumulative incidence function between patients with non variceal bleeding and age, sex, year, and general practice matched controls without non variceal bleeding. * Confidence intervals were too unstable to be interpretable for respiratory causes of death due to small numbers.
